# Interventricular septum involvement is related to right ventricular dysfunction in anterior STEMI patients without right ventricular infarction: a cardiovascular magnetic resonance study

**DOI:** 10.1007/s10554-024-03166-z

**Published:** 2024-07-22

**Authors:** Shichu Liang, Shi Chen, Yanlin Bai, Min Ma, Fanfan Shi, Litao Huang, Hua Wang, Chunchao Xia, Kaiyue Diao, Yong He

**Affiliations:** 1 Department of Cardiology, West China Hospital, Sichuan University, No.37 GuoXue Alley, Chengdu, 610041 China; 2 West China School of Medicine, West China Hospital, Sichuan University, Chengdu, Sichuan China; 3 Department of Clinical Research and Management, Center of Biostatistics, Design, Measurement and Evaluation (CBDME), West China Hospital, Sichuan University, Chengdu, China; 4 Department of Radiology, West China Hospital, Sichuan University, No.37 GuoXue Alley, Chengdu, 610041 China

**Keywords:** Cardiovascular magnetic resonance, Anterior ST-segment elevation myocardial infarction, Right ventricular dysfunction, Interventricular septum

## Abstract

The value of cardiovascular magnetic resonance (CMR) in assessing and predicting acute right ventricular (RV) dysfunction in patients with anterior ST-segment elevation myocardial infarction (STEMI) remains ascertained. Eighty eight patients with anterior STEMI were prospectively recruited and underwent CMR examinations within one week following the coronary intervention. Patients with RV ejection fraction (RVEF) less than 2 standard deviations below the average at the center (RVEF ≤ 45.0%) were defined as having RV dysfunction. The size of infarction, segmental wall motion, and T1 and T2 mapping values of global myocardium and the interventricular septum (IVS) were measured. Predictive performance was calculated using receiver-operating characteristic curve analysis and logistic regression test. Twenty two patients presented with RV dysfunction. The RV dysfunction group had a larger IVS infarct extent (54.28 ± 10.35 vs 33.95 ± 15.09%, P < 0.001) and lower left ventricle stroke volume index (33.93 ± 7.96 vs 42.46 ± 8.14 ml/m^2^, P < 0.001) compared to the non-RV dysfunction group. IVS infarct extent at 48.8% best predicted the presence of RV dysfunction with an area under the curve of 0.864. Left ventricular stroke volume index (LVSVI) and IVS infarct extent were selected by stepwise multivariable logistic regression analysis. Lower LVSVI (odds ratio [OR] 0.90; 95% confidence interval [CI], 0.79 to 0.99; P = 0.044) and higher IVS infarct extent (OR 1.16; 95% CI 1.05 to 1.33; P = 0.01) were found to be independent predictors for RV dysfunction. In patients with anterior STEMI, those with larger IVS infarct extent and worse LV function are more likely to be associated with RV dysfunction.

**Supplementary Information:**

The online version contains supplementary material available at 10.1007/s10554-024-03166-z.

## Introduction

Anterior ST-segment elevation myocardial infarction (STEMI), caused by the occlusion of the left anterior descending (LAD) artery, is associated with a poor prognosis due to a large extent of myocardial damage [1]. Although the infarction of the anterior wall results in left ventricular (LV) dysfunction, the focus on LV dysfunction has often led to overlooking right ventricular (RV) dysfunction. RV dysfunction has been identified as a risk factor for heart failure and mortality in patients with STEMI [2]. Previous studies have noted that RV is more tolerant to ischemia than LV is, but the prevalence of RV dysfunction has been reported to vary widely from studies. An autopsy study by Andersen et al. [3]. found that 84% of hearts with coronary heart disease had RV scars. While RV function is a predictor of post-infarction adverse events [4, 5], accurately sizing and calculating the function of the RV is difficult to achieve via echocardiography owing to its complex geometry [6, 7]. Therefore, it would be beneficial to identify additional indicators of RV injury and predictors of RV dysfunction to better evaluate RV involvement in patients experiencing anterior STEMI.

Studies have demonstrated that LV dysfunction is closely correlated with the presence of RV dysfunction [8, 9]. The interaction between the two ventricles through the interventricular septum (IVS) has been proposed as a possible explanation for this interrelated injury, particularly in patients with anterior myocardial infarction [10]. Therefore, LV parameters could be useful in alerting healthcare providers about the presence of RV dysfunction. Furthermore, significant advancements have been made in quantifying biventricular function, and cardiovascular magnetic resonance (CMR) is widely recognized as the gold standard for noninvasive assessment of cardiac function, anatomy, and myocardial characterization. Compared to echocardiography, CMR is superior with regard to the ability to accurately visualize the endo- and epicardium [11, 12], providing a more precise tool for quantifying RV function and risk stratification [13]. Additionally, multiple CMR sequences can be used to characterize LV tissue, with proven correlation to myocardial pathology results [14]. CMR can also detect subclinical changes in patients with preserved LV ejection fraction (LVEF) and predict the prognosis for patients with cardiomyopathy [15].

The relationship between RV and LV morphology is complex [7], and the correlation between CMR-derived LV parameters and RV dysfunction has not been systematically investigated heretofore. The aim of this study is to utilize CMR technology to investigate the occurrence, clinical characteristics, and clinical correlates of RV dysfunction in patients with acute anterior STEMI.

## Materials and methods

### Study group

This study was conducted in accordance with the Declaration of Helsinki and approved by the Institutional Review Board of West China Hospital, Sichuan University. All participants provided written informed consent prior to their inclusion in the study. The confidentiality of personal data was maintained and only used for the purpose of this trial.

Data were prospectively collected from our center from September 2015 to March 2017. A total of 121 consecutive patients referred to the chest pain center were initially recruited for this study. Patients who were diagnosed with first-time anterior STEMI based on the STEMI clinical guidelines [16] and received primary percutaneous coronary intervention (PPCI) were included in the study. The culprit infarct-related artery was the LAD. Successful PCI was defined as a residual stenosis of less than 20% and thrombolysis in myocardial infarction (TIMI) grade 3 flow, without any serious complications.

All patients included in the study underwent CMR examination within one week after PPCI. The exclusion criteria were as follows: (1) RV infarction, as defined by an ST-segment elevation of 0.1 mV or greater in leads V3R and V4R on ECG at presentation [16] and/or any RV wall motion abnormality detected by echocardiography; (2) previous coronary artery bypass surgery or revascularization intervention; (3) any cardiomyopathy, valvular disease, or congenital heart disease; (4) severe diseases that independently affect RV function, such as old inferior wall infarction, old RV infarction, severe chronic obstructive pulmonary disease, interstitial lung disease, pulmonary embolism, and primary pulmonary hypertension; (5) contraindication to CMR (e.g., pacemaker and claustrophobia) or insufficient image quality; (6) pregnancy; (7) inability to lie in a supine position; (8) an estimated glomerular filtration rate of 30 ml/(min·1.73 m^2^) or less; (9) patients with significant stenosis (> 50%) affecting RV branch or right coronary artery (RCA) proximal to RV branch; and (10) consent refusal.

Basic characteristics such as age, sex, body mass index (BMI), and body surface area (BSA), as well as cardiovascular risk factors like smoking history, underlying diseases including hypertension, diabetes mellitus, hypercholesterolemia, and relevant laboratory findings (triglyceride, cholesterol, serum creatinine, cardiac troponin [cTnT] peak, B-type natriuretic peptide [BNP] peak, high-density lipoprotein cholesterol [HDL-C], low-density lipoprotein cholesterol [LDL-C], white blood cell [WBC] count, and platelet [PLT] count) were collected from the patient’s history recordings. Peri-PPCI variables including pre- and post- TIMI flow, pain to device time (time to PCI-mediated reperfusion), and whether thrombectomy was performed were also recorded. BSA was calculated using the formula: BSA (m^2^) = 0.0061 × height (cm) + 0.0128 × weight (kg) − 0.1529.

### Magnetic resonance imaging

CMR examinations were conducted using a 3.0 T MR scanner (MAGNETOM Skyra; Siemens Healthcare, Erlangen, Germany). The examinations were performed by an MRI trainee technician, who was supervised by a senior technician with at least 5 years of experience in CMR imaging. The patients were placed in a supine position, and an 18-element body phased array coil was used during the examination. A standard electrocardiographic triggering device was used for heart rate triggering and monitoring.

A balance steady-state free precession sequence was used to acquire contiguous short-axis (SAX) slices encompassing the whole LV, as well as standard two-, three-, and four-chamber long-axis (LAX) cine images during repeated breath holds. The parameters for this sequence were: repetition time (TR), 40.25 ms; echo time (TE), 1.2 ms; flip angle, 40°; field of view (FOV), 340 × 285 mm^2^; matrix, 208 × 139; slice thickness, 8 mm; and number of phases, 25. T2 mapping was acquired with a T2-prepared steady-state free precession sequence, with the following parameters: TR, 272.95 ms; TE, 1.06 ms; flip angle, 35°; matrix, 192 × 116, FOV: 360 × 289 mm^2^; and slice thickness, 8 mm. Native T1 mapping was performed using a modified look-locker inversion recovery sequence, with the following parameters: TR, 319.83 ms; TE, 1.17 ms; flip angle, 35°; matrix, 256 × 145; field of view, 360 × 307 mm^2^; and slice thickness, 8 mm. A 5s(3s)3s modified sampling protocol was used. Standard SAX slices at apical, middle, and basal level of the LV, as well as two-, three-, and four-chamber colored native T1 and T2 maps, were generated after motion correction of the set of images acquired at different inversion times. A dose of 0.1 mmol/kg gadolinium (gadodiamide, 469.01 mg/ml; gadopentetic acid dimeglumine salt injection, Bayer, Germany) was injected at a flow rate of 2.5–3.0 ml/s. Late gadolinium enhancement (LGE) images were then acquired 10–15 min after contrast administration using a phase-sensitive inversion recovery sequence, with the following parameters: TR, 824 ms; TE, 3.5 ms; flip angle, 40°; matrix, 256 × 127, FOV, 400 × 275 mm; and slice thickness, 8 mm.

### Image post-processing

Image post-processing and analysis were performed by two radiologists who were blinded to the clinical data, using commercially available software (Cvi42; Circle Cardiovascular Imaging, Inc., Calgary, Canada). In case of any discrepancy or disagreement between the two radiologists, a third senior radiologist with over 10 years of experience was consulted. Global cardiac function indexes, including LV/RV end-diastolic volume (EDV) and end-systolic volume (ESV), as well as ejection fraction (EF), were analyzed in the SAX view following the standardized protocol of Society of Cardiovascular Magnetic Resonance post-processing guideline [17]. LV/RV stroke volume (SV) was calculated as LV/RV EDV-LV/RV ESV. The LV/RV EDV index (EDVI), ESV index (ESVI) and SV index (SVI) was calculated as LV/RV EDV, ESV and SV/BSA. RV dysfunction was defined as RV ejection fraction (RVEF) of less than 45% [18].

**Fig. 1:**
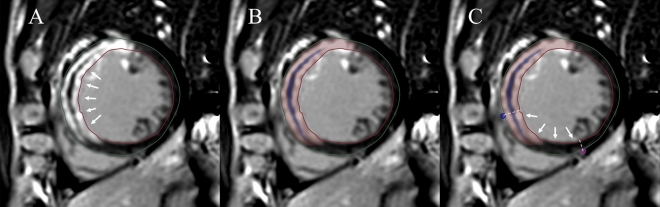
Post-processing of the CMR images. **A** The high signal intensity in the left ventricular anterior wall and the IVS on LGE imaging indicates infarction area, while the low signal intensity (white arrows) inside the infarction area indicates MVO. **B** Exhibited the infarction area and MVO using red and blue color. The infarct extent is calculated as the area of red and blue as a percentage of the total myocardial mass. **C** The area (white arrows) between the anterior (blue point) and posterior (red point) right ventricle insertion points indicates IVS. The infarction area as demonstrated by the red and blue color inside IVS was recorded as the infarction size in IVS (CMR cardiovascular magnetic resonance, LGE late gadolinium enhancement, MVO microvascular obstruction, IVS interventricular septum)

Subsequently, the infarction area was defined on the LGE SAX images as an area with a signal intensity greater than 5 standard deviations (SD) above the average intensity of the region of interest (ROI) drawn at the remote normal myocardium [19]. Microvascular obstruction (MVO) area was determined by visually recognizing the core area inside the infarcted myocardium without enhancement on the baseline LGE images. The overall size and volume of IVS were outlined and analyzed separately, with an extra ROI placed to calculate the infarction size at the IVS region (Fig. 1). The native T1 and T2 of the IVS were measured. End-diastolic wall thickness (EDWT) and end-systolic wall thickness (ESWT) were calculated by referring to the 16-AHA segmentation method. Systolic wall thickening was calculated by using the formula: (ESWT − EDWT)/EDWT × 100%.

Myocardial strain analysis was performed in a semi-automated manner using the Cvi42 5.12.1 software (Tissue Tracking, Circle Cardiovascular Imaging Inc., CVI42® 5.12.1 software, Calgary, Alberta). A stack of SAX images between the LV apex and the mitral valve plane, as well as two- and four-chamber LAX cine images were used for the analysis. Endocardial and epicardial borders were manually contoured, and both the anterior and posterior RV insertion points were manually determined at the end-diastolic phase. In addition, 2D global longitudinal strain (GLS) was acquired from the two LAX slices, while 2D global circumferential strain (GCS) and 2D global radial strain (GRS) were acquired from the SAX slices. The strain analysis was performed by the software in a semi-automated manner.

### Statistical analysis

The patients were divided into two groups, RV dysfunction and non-RV dysfunction, to compare the CMR data. Continuous measures were compared using either a two-sided non-paired Student’s *t*-test or Mann–Whitney *U*-test as appropriate. Categorical measures were compared using a chi-square test. Pearson’s linear regression analyses were used to determine the relationships between RVEF and LV CMR variables. The linear regression coefficients (beta), correlation coefficients, and P-values were reported. Optimal cut-off values for predicting RV dysfunction with CMR indexes were determined by receiver operating characteristic (ROC) curve analysis, where sensitivity and specificity intersected. Univariate logistic regression analysis was used to test the associations between LV CMR variables and RV dysfunction. Stepwise multiple logistic regression analysis was performed to identify clinical and imaging variables associated with RV dysfunction. The optimal threshold was determined by Youden index (Youden index = sensitivity + specificity − 1) [20]. Variables with a P-value of less than 0.05 on bivariable analysis were considered candidate variables in the multivariate logistic regression model. In all analyses, a P-value of less than 0.05 was considered statistically significant, and all the analyses were performed using the R project V 3.3.1 (R Project for Statistical Computing, Vienna, Austria).

## Results

### Clinical characteristics of the study population

**Fig. 2:**
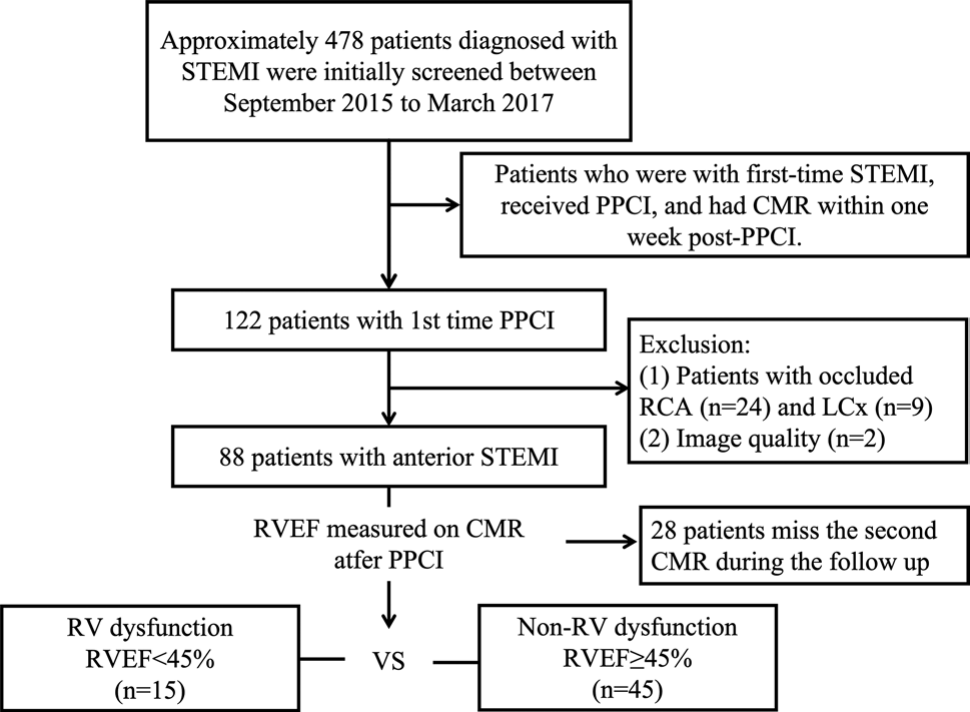
Study protocol. (*STEMI* ST-segment elevation myocardial infarction, *PPCI* primary percutaneous coronary intervention, *CMR* cardiovascular magnetic resonance, *RCA* right coronary artery, *LCx* left circumflex artery, RV right ventricular, *RVEF* right ventricular ejection fraction)

Out of the 122 patients with PPCI who were included in the study, 24 and nine were excluded due to occlusion of the RCA and left circumflex (LCx) arteries, respectively. One patient was excluded from the study due to unsatisfactory image quality. Finally, 88 patients [age: 57.34 ± 11.18 years; males/females: 80/8] were included in the analysis (Fig. 2). Among the included patients, 77 (87.5%) were current smokers. Additionally, 41 out of 88 (46.6%) had hypertension, and 19 (21.6%) had diabetes mellitus (Table 1). The majority of the patients (n = 83, 94.3%) presented with a TIMI flow grade of 0 or I before the intervention, and all patients achieved a TIMI flow grade of III after the intervention. None of the patients died during hospitalization.

**Table 1:** Participants’ clinical characteristics

	All patients (n = 88)	RV dysfunction (n = 22)	Non-RV dysfunction (n = 66)	P value
Age (year)	57.34 ± 11.18	55.86 ± 8.68	57.83 ± 11.92	0.477
Male (%)	80 (90.9)	20 (90.9)	60 (90.9)	1
BMI (kg/m^2^)	24.07 [21.84, 26.14]	24.50 [22.07, 28.00]	23.82 [21.81, 26.02]	0.488
BSA (m^2^)	1.71 [1.62, 1.84]	1.67 [1.62, 1.90]	1.71 [1.63, 1.83]	0.992
Blood pressure, mm Hg				
Systolic	124.98 ± 21.60	128.36 ± 22.46	123.85 ± 21.37	0.399
Diastolic	78.43 ± 15.16	81.77 ± 19.18	77.32 ± 13.55	0.235
Heart rate, /min	80.00 [71.75, 89.00]	90.00 [76.25, 100.75]	76.00 [67.25, 85.00]	0.002*
Cardiovascular risk factors				
Current smoker (%)	77 (87.5)	18 (81.8)	59 (89.4)	0.577
Hypertension (%)	41 (46.6)	9 (40.9)	32 (48.5)	0.711
Hypercholesterolemia (%)	32 (36.4)	9 (40.9)	23 (34.8)	0.798
Diabetes mellitus (%)	19 (21.6)	4 (18.2)	15 (22.7)	0.881
Pain to device time, hours	6.00 [4.00, 10.00]	4.50 [3.25, 7.50]	6.00 [4.00, 11.00]	0.077
Pre-PCI TIMI flow grade (%)				0.017*
TIMI flow 0	52 (59.1)	19 (86.4)	33 (50.0)	
TIMI flow I	31 (35.2)	2 ( 9.1)	29 (43.9)	
TIMI flow II	3 ( 3.4)	1 ( 4.5)	2 ( 3.0)	
TIMI flow III	2 ( 2.3)	0 ( 0.0)	2 ( 3.0)	
Thrombectomy (%)	24 (27.3)	8 (36.4)	16 (24.2)	0.407
Stent number	1.00 [1.00, 1.00]	1.00 [1.00, 1.00]	1.00 [1.00, 1.00]	0.719
Laboratory findings				
Triglyceride (mmol/l**)**	1.39 [1.08, 2.09]	1.42 [1.10, 2.23]	1.39 [1.08, 2.05]	0.7
Cholesterol (mmol/l**)**	4.28 [3.80, 5.04]	3.93 [3.66, 4.57]	4.34 [3.81, 5.06]	0.347
HDL-C (mmol/l**)**	1.14 ± 0.29	1.12 ± 0.31	1.15 ± 0.29	0.75
LDL-C (mmol/l**)**	2.65 ± 0.95	2.40 ± 1.04	2.74 ± 0.91	0.143
Serum creatinine (μmol/l**)**	75.00 [67.00, 86.25]	73.00 [68.25, 87.00]	75.00 [67.00, 85.00]	0.896
BNP peak (pg/ml**)**	1599.00 [864.75, 3240.00]	2734.50 [1207.25, 3305.75]	1401.50 [796.50, 2512.00]	0.056
cTnT peak (ng/l)	5570.50 [2389.75, 8332.00]	8036.50 [5577.25, 10,001.00]	4588.00 [1875.50, 6781.00]	< 0.001*
WBC (10^9^/l)	9.69 ± 3.00	9.40 ± 2.71	9.78 ± 3.10	0.606
PLT (10^9^/l)	169.00 [139.00, 208.50]	152.50 [137.50, 204.25]	175.00 [141.50, 209.25]	0.328

*BMI* body mass index, *PCI* percutaneous coronary intervention, *TIMI* thrombolysis in myocardial infarction, *HDL-C* high-density lipoprotein cholesterol, *LDL-C* low-density lipoprotein cholesterol, *BNP* B-type natriuretic peptide, *cTnT* troponin T, *WBC* white blood cells, *PLT* platelet

*Statistical significance

A total of 22 out of 88 (25%) patients presented with RV dysfunction. Patients with RV dysfunction had slightly higher peak BNP levels (2734.50 [1207.25, 3305.75] pg/ml vs 1401.50 [796.50, 2512.00], P = 0.056) and significantly higher peak cTnT levels (8036.50 [5577.25, 10001.00] vs 4588.00 [1875.50, 6781.00], P < 0.001) than patients without RV dysfunction. Additionally, heart rate was significantly higher for patients with RV dysfunction than for patients without the same (90.00 [76.25, 100.75] vs 76.00 [67.25, 85.00], P = 0.002). A poor pre-PCI TIMI flow grade was found in patients with RV dysfunction. However, no prominent difference was found between the two groups concerning cardiovascular risk factors and PPCI-related variables (Table 1).

### CMR characteristics between the patients with and without RV dysfunction

In the RV dysfunction group, a total of 21 out of 22 (95.5%) patients presented with MVO, which was significantly higher than that in the non-RV dysfunction group (95.5% vs 50.0%, P < 0.001). Patients with RV dysfunction had a significantly larger infarction extent (45.40% ± 14.63% vs 29.03% ± 14.37%, P < 0.001), reduced LVEF (39.17% ± 10.61% vs 49.64% ± 9.29%, P < 0.001), lower LVSVI (33.93 ± 7.96 ml/m^2^ vs 42.46 ± 8.14 ml/m^2^, P < 0.001), as well as decreased GLS [− 9.11 (− 10.39, − 7.07)% vs − 11.15 (− 12.24, − 9.18)%, P = 0.006], GCS (− 10.73% ± 3.45% vs − 14.18% ± 3.32%, P = 0.0010), and GRS [13.09 (11.65, 18.24)% vs 19.28 (15.17, 22.70)%, P < 0.001]. Between-group differences were found in RVEDVI and RVSVI but not RVESVI (Table 2).

**Table 2:** Cardiovascular magnetic resonance characteristics for patients with and without RV dysfunction (n = 88)

	All patients (n = 88)	RV dysfunction (n = 22)	Non-RV dysfunction (n = 66)	P value
LGE size, g	37.42 [22.95, 52.41]	55.85 [40.23, 68.82]	27.92 [18.56, 44.45]	< 0.001*
LV mass, g	108.14 [93.82, 133.94]	124.14 [96.91, 144.08]	105.47 [93.71, 125.90]	0.056
Global LGE percentage (%)	33.13 ± 16.02	45.40 ± 14.63	29.03 ± 14.37	< 0.001*
MVO, n (%)	54 (61.4)	21 (95.5)	33 (50.0)	< 0.001*
RV function and strain indexes				
RVEF, %	53.45 [45.00, 61.92]	41.80 [37.02, 42.95]	58.67 [51.35, 64.11]	< 0.001*
RVESVI, ml/m^2^	59.39 ± 16.16	59.89 ± 18.21	59.22 ± 15.56	0.867
RVEDVI, ml/m^2^	26.21 [19.82, 33.49]	33.87 [28.90, 42.07]	23.62 [17.80, 30.41]	< 0.001*
RVSVI, ml/m^2^	31.83 ± 10.37	23.13 ± 6.71	34.73 ± 9.75	< 0.001*
LV function and strain indexes				
LVEF, %	47.02 ± 10.60	39.17 ± 10.61	49.64 ± 9.29	< 0.001*
LVESVI, ml/m^2^	85.53 [76.85, 96.00]	87.17 [78.47, 99.62]	84.77 [76.75, 95.80]	0.563
LVEDVI, ml/m^2^	44.12 [35.86, 56.53]	48.49 [42.13, 69.47]	43.04 [33.20, 52.62]	0.018*
LVSVI, ml/m^2^	40.33 ± 8.87	33.93 ± 7.96	42.46 ± 8.14	< 0.001*
LV-GLS, %	- 10.52 [ -12.09, -8.83]	- 9.11 [-10.39, -7.07]	- 11.15 [ -12.24, -9.18]	0.006*
LV-GCS, %	- 13.32 ± 3.66	- 10.73 ± 3.45	- 14.18 ± 3.32	< 0.001*
LV-GRS, %	17.60 [13.41, 21.63]	13.09 [11.65, 18.24]	19.28 [15.17, 22.70]	< 0.001*
IVS indexes				
Infarction mass, g	51.58 ± 13.32	60.54 ± 14.98	48.60 ± 11.35	< 0.001*
Infarction size, ml	46.89 [40.44, 55.43]	53.48 [49.17, 69.06]	45.16 [39.14, 54.24]	0.001*
IVS mass, g	21.09 ± 11.82	33.04 ± 11.36	17.11 ± 9.00	< 0.001*
IVS size, ml	20.09 ± 11.26	31.47 ± 10.82	16.30 ± 8.57	< 0.001*
Infarct extent, %	39.04 ± 16.56	54.28 ± 10.35	33.95 ± 15.09	< 0.001*
EDWT, mm	9.25 [7.41, 11.38]	10.38 [8.65, 11.69]	8.59 [7.19, 10.93]	0.056
ESWT, mm	9.97 ± 2.23	9.76 ± 2.44	10.04 ± 2.17	0.607
SWT, mm	47.91 [33.11, 61.13]	37.92 [26.08, 49.14]	51.77 [37.98, 62.63]	0.015*
IVS native T1, ms	1418.44 ± 80.98	1395.55 ± 71.98	1426.07 ± 82.86	0.126
IVS T2, ms	46.71 [42.88, 48.20]	43.98 [41.05, 46.61]	46.79 [44.14, 49.01]	0.006*

*LGE* late gadolinium enhancement, *MVO* microvascular obstruction, *RVEF* right ventricle ejection fraction, *RVESVI* right ventricle end-systolic volume index, *RVEDVI* right ventricle end-diastolic volume index, *RVSVI* right ventricle stroke volume index, *LVEF* left ventricle ejection fraction, *LVESVI* left ventricle end-systolic volume index, *LVEDVI* left ventricle end-diastolic volume index, *LVSVI* left ventricle stroke volume index, *LVGLS* left ventricle global longitudinal strain, *LVGCS* left ventricle global circumferential strain, *LVGRS* left ventricle global radial strain, *IVS* interventricular septum, *EDWT* end-diastolic wall thickness, *ESWT* end-systolic wall thickness, *SWT* systolic wall thickening

*Statistical significance

Regarding the IVS measurements, patients with RV dysfunction had a larger infarction area in the IVS region (all P < 0.01) than in the non-IVS region. Additionally, significantly lower T2 [43.98 (41.05, 46.61) vs 46.79 (44.14, 49.01), P = 0.006] was observed in patients with RV dysfunction compared with those without it, while the native T1 was not significant (1395.55 ± 71.98 vs 1426.07 ± 82.862, P = 0.0063). No significant differences existed in any of the IVS’s EDWT and ESDW between patients with RV dysfunction and those without the same. However, systolic wall thickening was lower in patients with RV dysfunction than in those without it [37.92 (26.08, 49.14) % vs 51.77 (37.98, 62.63) %, P = 0.015].

### Correlations of RV dysfunction with LV CMR variables

The cTnT peak, presence of MVO, global LGE percentage, LVEF, LVSVI, LV-GCS and GRS, IVS’s infarction size, and IVS T2 were all found to be associated with RV dysfunction. However, only LVSVI (odds ratio [OR], 0.90 [95% CI 0.79 to 0.99; P = 0.044) and IVS’s infarct extent (OR 1.16 [95% CI 1.05 to 1.33; P = 0.01) were identified as independent predictors for RV dysfunction (Table 3).

**Table 3:** Results of univariate and multivariate logistic regression analysis for predicting RV function

Clinical an imaging variable	Univariate logistic regression			Multiple logistic regression		
	OR	95% CI	P value	OR	95% CI	P value
Age (year)	0.98	0.94–1.03	0.473			
BMI (kg/m^2^)	1.04	0.92–1.18	0.5621			
cTnT peak (ng/l)	1.00	1.00–1.00	0.0009*			
BNP peak (pg/ml)	1.00	1.00–1.00	0.4652			
MVO, n (%)	21	2.67–165.32	0.0038*			
Global LGE percentage (%)	1.08	1.04–1.13	0.0003*			
LVEF, %	0.9	0.85–0.95	< 0.001*			
LVSVI, ml/m^2^	0.87	0.81–0.94	0.0004*	0.90	0.79–0.99	0.044
LVGLS, %	1.06	0.96–1.16	0.2513			
LVGRS, %	0.84	0.76–0.93	0.0013*			
LVGCS, %	1.36	1.14–1.62	< 0.001*			
IVS infarct extent, %	1.14	1.07–1.22	< 0.001*	1.16	1.05–1.33	0.010
IVS Native T1, ms	1	0.99–1	0.1294			
IVS T2, ms	0.87	0.77–0.98	0.0277*			

*OR* Odds ratio, *BMI* body mass index, *LGE* late gadolinium enhancement, *MVO* microvascular obstruction, *LVEF* left ventricle ejection fraction, *LVESVI* left ventricle end-systolic volume index, *LVGCS* left ventricle global circumferential strain, *LVGRS* left ventricle global radial strain, *IVS* interventricular septum

*Statistical significance

**Fig. 3:**
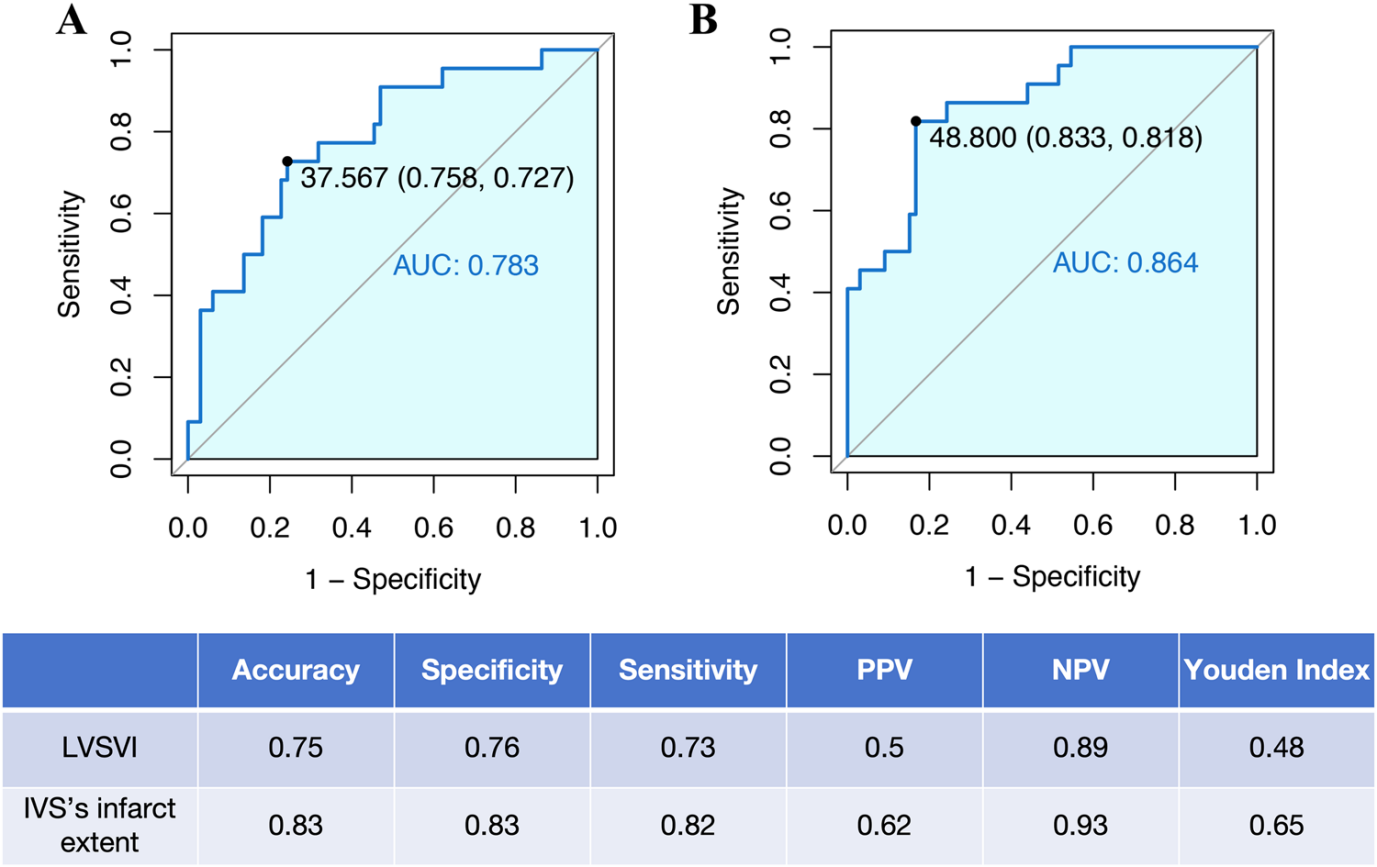
ROC curves for LVSVI and IVS’s infarct extent for predicting right ventricular dysfunction (*LVSVI* left ventricular stroke volume index, *IVS* interventricular septum, *AUC* area under the curve, *PPV* positive predictive value, *NPV* negative predictive value)

The diagnostic accuracy of RV dysfunction using different CMR variables is presented in Fig. 3. The single cut-off that maximised sensitivity and specificity (Youdens index) for LVSVI was 37.567 ml/m^2^ (AUC, 0.783) and IVS infarct extent was 48.8% (AUC, 0.864) were found to be the best predictors of RV dysfunction during the acute phase. The positive predictive value (PPV) and negative predictive value (NPV) at Youdens index for LVSVI were 50.0% and 89.2%, respectively, whilst the PPV and NPV for IVS infarct extent were 62.1% and 93.2%, respectively.

## Discussion

Patients with anterior STEMI combined with RV dysfunction are at a higher risk of in-hospital and long-term mortality [5, 21]. Anterior STEMI with RV involvement may have its clinical manifestations obscured by LV dysfunction. Inappropriate management can precipitate severe hemodynamic derangements, potentially culminating in mortality. Early identification of RV function amidst LV ischemia post-myocardial infarction holds considerable significance for guiding clinical diagnosis, therapeutic interventions, and prognostic evaluation. Our study showed that approximately one-fourth of patients with anterior STEMI had RV dysfunction. To our knowledge, this is the first CMR study that has demonstrated the correlation between RV dysfunction and CMR imaging parameters of LV and IVS for patients with anterior STEMI. Our results suggest that LV dysfunction accompanied by primarily IVS-related infarction might indicate RV dysfunction and should be considered a potential risk factor for RV dysfunction when assessing patients with anterior STEMI.

The percentage of patients presenting with RV abnormalities in our study was 25%, which is within the range reported by previous studies [18]. Masci et al. [22] reported the highest rate at approximately 50%, when measured by myocardial edema. In contrast, we defined RV dysfunction based only on RVEF and reported a lower rate of RV dysfunction. However, we assumed that more patients may have RV injury but no significant functional abnormality. Matthias et al. [23] reported a similar rate of RV dysfunction at 16.5%, for which the definition of RV injury required both myocardial edema and reduced wall motion. Given the irregular geometry of the RV and measurement variance, it has been difficult to reach a common consensus on the definition of RV dysfunction. A review reported less than 45% as the incidence of RV dysfunction in patients with non-ischemic disease [24, 25], while no definite cut-off for patients with ischemic diseases has been determined. In this study, we used RVEF < 45% as the standard to avoid measurement variance from other centers. RV dysfunction defined using RVEF has been proven to be associated with adverse clinical outcomes for patients with ischemic heart disease [26].However, the clinical significance of subclinical RV dysfunction in the absence of a reduced RVEF necessitates further validation [27]. RV strain could potentially serve as a valuable tool in aiding our understanding of this condition.

**Fig. 4:**
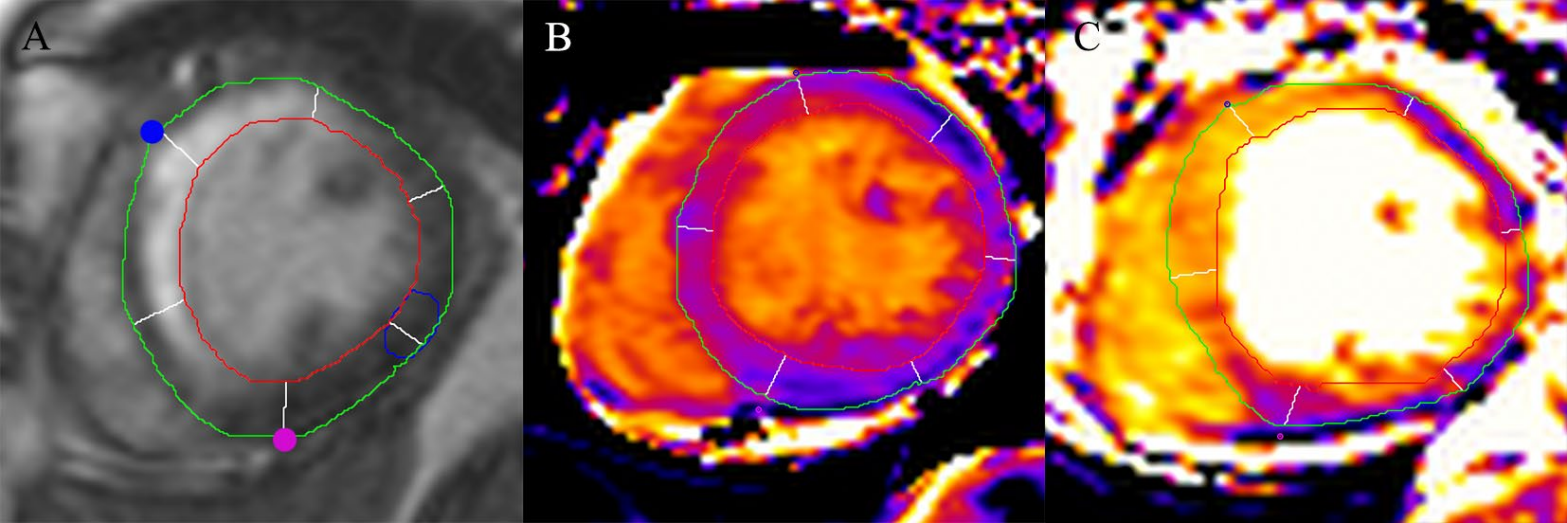
Case presentation: A 67-year old female with left anterior descending artery occlusion. **A** Infarction at the anterior and interventricular septum (IVS) was detected by late gadolinium enhancement (black arrows). The anterior (blue) and posterior (red) right ventricle insertion points were manually marked. The myocardium was then divided into six segments for the middle layer. **B** Native T1 mapping showed higher intensity at IVS (black arrow). **C** T2 mapping showed higher intensity at IVS and the anterior wall (black arrows)

We found that patients with RV dysfunction had a larger reduction in LVEF and LVSVI, more infarction size at IVS, and a higher frequency of MVO. Consistent with an echocardiographic study [21], all patients with RV dysfunction in our study presented with LV dysfunction, except for one patient without the presence of MVO but with a large involvement of the IVS (54.4% area of the whole IVS; Fig. 4 and Supplemental Fig. 1). Tricuspid annular plane systolic excursion, a quantitative index of RV systolic function, has been demonstrated to positively correlate with LVEF in an echocardiographic study, implying that a reduction in LVEF may adversely affect RV systolic function [5, 21]. Another study also found that impaired LV diastolic function resulting from anterior STEMI contributes to RV dysfunction six months later [28].

LGE imaging can provide additional prognostic information in patients with myocardial infarction [29, 30]. In our study, we found that patients with RV dysfunction had a larger infarction size of the whole heart. However, our regression analysis indicated that the global LGE percentage was not statistically significant in the multivariable analysis. The result demonstrated a positive correlation between IVS involvement and the presence of RV dysfunction, suggesting a joint role that IVS potentially plays in the interaction between RV with LV [23]. IVS is the “engine” of RV, as the torsions of the IVS help RV ejection, and the alternating pattern of torsions helps the rapid filling of RV. The left coronary artery is tasked with supplying the anterior two-thirds of the myocardium in the IVS. Consequently, when the left coronary artery is stenosed, the blood supply to the anterior septum diminishes, impairing the IVS’s mobility and, as a result, compromising the overall functional capacity of the RV. Our study contributes to this understanding by demonstrating that IVS’s T1 and T2 mapping values, rather than wall motion indexes, were reduced in patients with RV dysfunction. Native T1 and T2 values have been observed to increase in myocardial inflammation and edema and to decrease in the presence of MVO for STEMI patients [31–33]. We believe that the reduction of T1 and T2 in IVS regions for patients with RV dysfunction reflects the presence of MVO. We further noted that within the cohort exhibiting RV dysfunction, who may have a greater extent of IVS infarction, the T2 values of the IVS were somewhat reduced. This reduction could be attributed to the potential occurrence of MVO in certain patients with extensive infarct size. MVO is characterized by a diminished signal on T2-weighted imaging, which corresponds with our observed reduction in T2 values for the IVS in patients with RV dysfunction [34]. Thus, our results suggest that it might be the severity of injury at IVS that affects RV function, rather than simply taking part in the RV myocardium movement.

Nevertheless, several limitations should be noted in our study. First, this was a single-center study with a limited number of patients. Therefore, further multiple center studies should be performed to validate the predictors of RV dysfunction. Second, neither of the finding of LV dysfunction or large IVS infarction size necessarily indicate RV dysfunction. The research undertaken thus far, along with the evidence gathered, has not reached the level of sufficiency required to conclusively establish a cause-and-effect relationship between LV dysfunction and RV dysfunction. The NPV was high (85.2–93.2%) while the PPV was relatively low. Thus, our study only suggests that LV dysfunction and large involvement of the IVS could be used as screening tool for alerting RV dysfunction. Other parameters should be closely measured and documented to decide if the patients would develop RV dysfunction. Third, parametric mapping measures were not performed for RV myocardium. Due to the thickness of RV myocardium and artifacts, it was difficult to ensure reproducibility in measuring native T1 and T2 values on RV myocardium. Therefore, this analysis was not performed in our study. In future studies, RV dysfunction should be assessed from multiple aspects rather than RVEF.

## Conclusion

RV dysfunction occurs in approximately one-fourth of patients with anterior STEMI. Patients with a larger IVS infarction and reduced LV function are more likely to be associated with RV dysfunction. Almost all cases of RV dysfunction are accompanied by LV dysfunction. However, the prognostic and clinical value of these associated factors in patients with RV dysfunction remains to be ascertained. Further studies are required to validate these findings and to explore the clinical implications of RV dysfunction in patients with anterior STEMI.

## Supplementary Information

Below is the link to the electronic supplementary material.

Supplementary file1 (TIF 97434 KB) The American Heart Association’s 16-segment plot for the Case presentation. (A) Native T1 mapping; (B) T2 mapping

## Data Availability

The datasets during and/or analyzed during the current study are available from the corresponding author on reasonable request.

## Abbreviations

CMR: Cardiovascular magnetic resonance
EDVI: End-diastolic volume index
EDWT: End-diastolic wall thickness
ESVI: End-systolic volume index
ESWT: End-systolic wall thickness
GCS: Global circumferential strain
GLS: Global longitudinal strain
GRS: Global radial strain
IVS: Interventricular septum
LAD: Left anterior descending
LGE: Late gadolinium enhancement
LV: Left ventricular
LVEF: LV ejection fraction
MVO: Microvascular obstruction
RV: Right ventricular
STEMI: ST-segment elevation myocardial infarction
TIMI: Thrombolysis in myocardial infarction
